# Alcohol Dependence Induces CRF Sensitivity in Female Central Amygdala GABA Synapses

**DOI:** 10.3390/ijms23147842

**Published:** 2022-07-16

**Authors:** Larry Rodriguez, Dean Kirson, Sarah A. Wolfe, Reesha R. Patel, Florence P. Varodayan, Angela E. Snyder, Pauravi J. Gandhi, Sophia Khom, Roman Vlkolinsky, Michal Bajo, Marisa Roberto

**Affiliations:** 1 Department of Molecular Medicine, The Scripps Research Institute, La Jolla, CA 92037, USA; lrodriguez@scripps.edu (L.R.); wolfe.sarah.a@gmail.com (S.A.W.); reesha.patel@me.com (R.R.P.); fvaroday@binghamton.edu (F.P.V.); asnyder@scripps.edu (A.E.S.); pgandhi@scripps.edu (P.J.G.); sophia.khom@univie.ac.at (S.K.); vlkolins@scripps.edu (R.V.); mbajo@scripps.edu (M.B.); 2 Department of Pharmacology, Addiction Science, and Toxicology, The University of Tennessee Health Science Center, Memphis, TN 38163, USA; 3 Department of Psychology, Binghamton University-SUNY, Binghamton, NY 13902, USA; 4 Department of Pharmaceutical Sciences, University of Vienna Josef-Holaubek-Platz 2, A-1090 Vienna, Austria

**Keywords:** corticotropin releasing factor (CRF), patch-clamp electrophysiology, sex difference, alcohol use disorder (AUD), Gamma-Aminobutyric Acid (GABA), central amygdala (CeA), spontaneous inhibitory postsynaptic currents (sIPSCs)

## Abstract

Alcohol use disorder (AUD) is a chronically relapsing disease characterized by loss of control in seeking and consuming alcohol (ethanol) driven by the recruitment of brain stress systems. However, AUD differs among the sexes: men are more likely to develop AUD, but women progress from casual to binge drinking and heavy alcohol use more quickly. The central amygdala (CeA) is a hub of stress and anxiety, with corticotropin-releasing factor (CRF)-CRF_1_ receptor and Gamma-Aminobutyric Acid (GABA)-ergic signaling dysregulation occurring in alcohol-dependent male rodents. However, we recently showed that GABAergic synapses in female rats are less sensitive to the acute effects of ethanol. Here, we used patch-clamp electrophysiology to examine the effects of alcohol dependence on the CRF modulation of rat CeA GABAergic transmission of both sexes. We found that GABAergic synapses of naïve female rats were unresponsive to CRF application compared to males, although alcohol dependence induced a similar CRF responsivity in both sexes. In situ hybridization revealed that females had fewer CeA neurons containing mRNA for the CRF_1_ receptor (*Crhr1*) than males, but in dependence, the percentage of *Crhr1*-expressing neurons in females increased, unlike in males. Overall, our data provide evidence for sexually dimorphic CeA CRF system effects on GABAergic synapses in dependence.

## 1. Introduction

Alcohol use disorder (AUD) is a chronically relapsing disorder characterized by a preoccupation with alcohol consumption, a loss of control in limiting intake, and the emergence of negative emotional states during withdrawal (also known as hyperkatifeia, which includes dysphoria, anxiety, irritability) [1,2]. Excessive alcohol consumption associated with negative emotional states observed in withdrawal occurs via negative reinforcement mechanisms such that alcohol alleviates the symptoms of withdrawal [3,4,5,6,7]. Thus, brain regions, such as the central nucleus of the amygdala (CeA), that are involved in processing stress, anxiety, and other withdrawal-associated states are recruited in the development of AUD. The CeA is a primarily Gamma-Aminobutyric Acid (GABA) -ergic nucleus that was shown to be involved in excessive alcohol consumption related to dependence and withdrawal, across species [8,9,10,11].

The CeA also contains many neuropeptide systems that modulate synaptic activity. The peptide corticotropin-releasing factor (CRF), a key mediator of stress responses, is locally produced and released by neurons within multiple brain regions, including the hypothalamus, the CeA, and other afferent regions [12]. CRF is a 41-residue polypeptide that binds to the G-protein-coupled CRF type 1 (CRF_1_) and CRF type 2 (CRF_2_) receptors [13,14]. While CRF produces its effects by binding to both receptors, it has a greater affinity for CRF_1_ [12,15]. The role of the CRF_1_ system was characterized in multiple rodent models of alcohol dependence. For example, ethanol withdrawal increased CRF levels in the amygdala of rats [16]. In alcohol-dependent rats, chronic treatment with a CRF_1_ antagonist blocked alcohol withdrawal-induced increases in alcohol drinking, and in non-dependent rats, the CRF_1_ antagonist tempered moderate increases in alcohol consumption [17]. In addition, the escalation of alcohol self-administration and anxiety typically observed during protracted abstinence can be blocked by competitive CRF_1_ antagonists [18,19,20,21].

Despite preclinical evidence suggesting that CRF_1_ antagonists would be efficacious in the treatment of alcohol dependence, clinical trials of CRF_1_ antagonist-based therapies to treat AUD in humans have shown mixed results [22,23]. Thus, more work is needed to fully understand the neuroadaptations that facilitate and sustain alcohol dependence, and in particular, the impact of sex on the underlying neurobiology of the disease. Potential sex differences are especially important considering the heightened activity of stress- and anxiety-related brain regions in alcohol dependence, and the inherent sex differences in stress responses [24,25]. Many neuropsychiatric disorders, including anxiety disorders and stress- and trauma-related disorders, differ by sex [26,27,28], and preclinical studies have shown differences in stress and anxiety processing between males and females [24]. More recently, transgenic reporter mice have been used to study sex differences in the CRF_1_ system [29], and while dependence was shown to sensitize females to the effects of acute alcohol [30], to our knowledge, no one has studied the neuroadaptations in the female CeA CRF system during alcohol dependence.

In this study, we induced alcohol dependence in rats using an established model of chronic intermittent ethanol vapor exposure and used whole-cell patch-clamp electrophysiology and in situ hybridization (ISH) to identify sex-specific neuroadaptations in the regulatory function of CRF at GABAergic synapses. Within the CeA of naïve and dependent rats of each sex, we determined the effects of the exogenous acute application of different concentrations of CRF and assessed tonic CRF_1_ receptor activity (using a selective CRF_1_ antagonist, R121919) on spontaneous inhibitory GABAergic postsynaptic currents (sIPSCs). We also determined the levels of mRNA co-expression of the CRF_1_ receptor with GABAergic neuronal markers in the CeA in both sexes.

## 2. Results

### 2.1. Sex Differences in Baseline sIPSC Kinetics of CeA GABA Synapses

**Figure 1:**
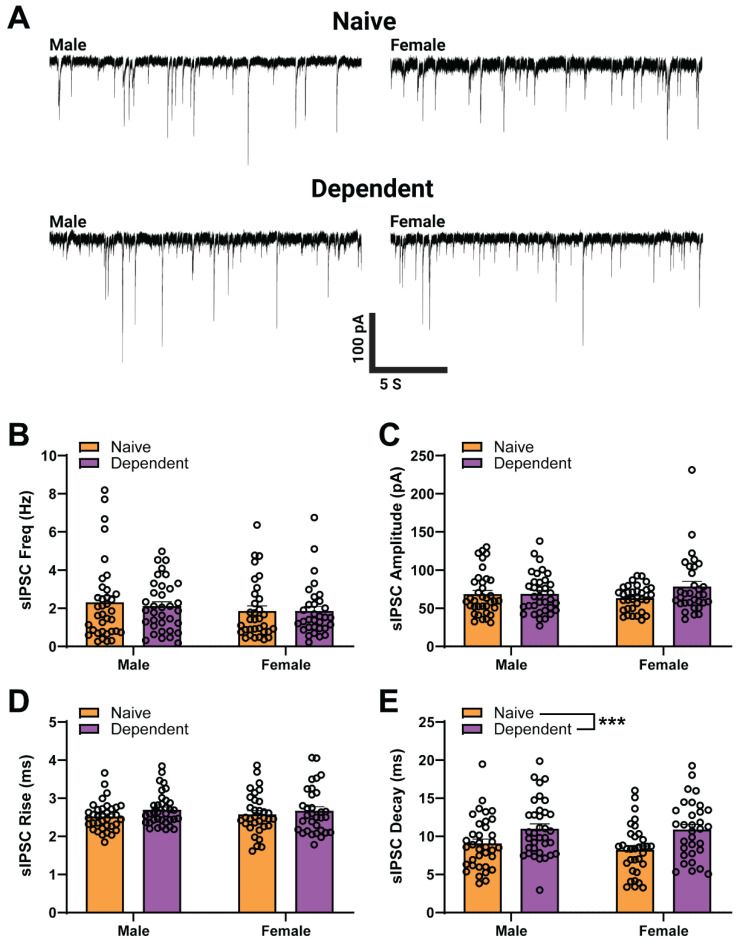
Baseline spontaneous GABAergic transmission in central amygdala (CeA) of males and females. (**A**–**E**): (**A**) Representative GABA_A_-mediated sIPSCs from CeA neurons of male (**left**) and female (**right**) rats, either naïve (**upper**) or alcohol dependent (**lower**). There is no effect of Sex or Alcohol Exposure and no interaction effect in CeA baseline sIPSC frequency (**B**), amplitude (**C**), or rise time (**D**). For sIPSC decay time (**E**), there is no main effect of Sex or interaction effect, but there is a main effect of Alcohol Exposure, primarily driven by dependent females with a prolonged decay time (10.88 ± 0.67 ms) compared to naïve females (8.24 ± 0.57 ms). Differences in baseline sIPSC properties are assessed using two-way ANOVA test.; Bars represent Mean ± SEM; *** denotes *p* < 0.001; *n* = 31–36 neurons per group; *N* = 19–25 rats per group.

**Figure A1:**
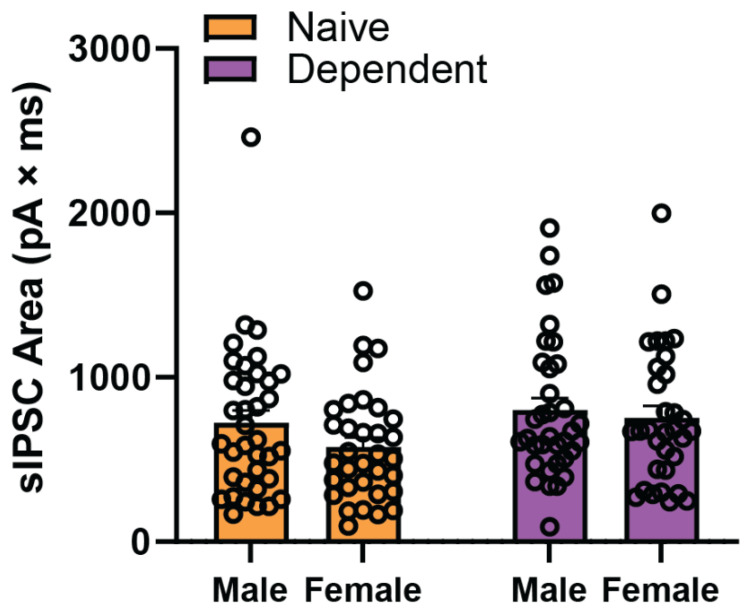
Integrated area of baseline spontaneous inhibitory postsynaptic currents in central amygdala (CeA) of alcohol naïve and alcohol dependent males and females. There were no differences in sIPSC area between the groups. Differences in baseline sIPSC properties are assessed using two-way ANOVA test.; Bars represent Mean ± SEM; *n* = 31–36 neurons per group; *N* = 19–25 rats per group.

We performed whole-cell patch-clamp recordings of GABAergic sIPSCs (Figure 1A) from neurons (*n* = 132) in the medial subdivision of the CeA of both male (left) and female (right), naïve (top) and alcohol-dependent (bottom) rats. In line with our previous results [30], here we found no main effect of sex or alcohol exposure on baseline sIPSC frequency (Figure 1B), amplitude (Figure 1C), or rise time (Figure 1D). Furthermore, our results revealed a main effect of alcohol exposure on sIPSC decay time (two-way ANOVA; Alcohol Exposure *F*_1,132_ = 14.2, *p* < 0.001) as dependent animal groups had higher decay times on average (10.94 ms) compared to naïve animal groups (8.66 ms) (as in [30]) (Figure 1E). Additionally, there was neither a main effect of Sex nor a Sex × Alcohol Exposure interaction effect, and no main effect of Sex or Alcohol Exposure on charge transfer (i.e., sIPSC area; see Figure A1 in Appendix A). These data suggest that alcohol dependence is altering postsynaptic GABA_A_ receptor function.

### 2.2. Alcohol Dependence Induces Responsivity of Female CeA GABAergic Synapses to Acute CRF Application

**Figure 2:**
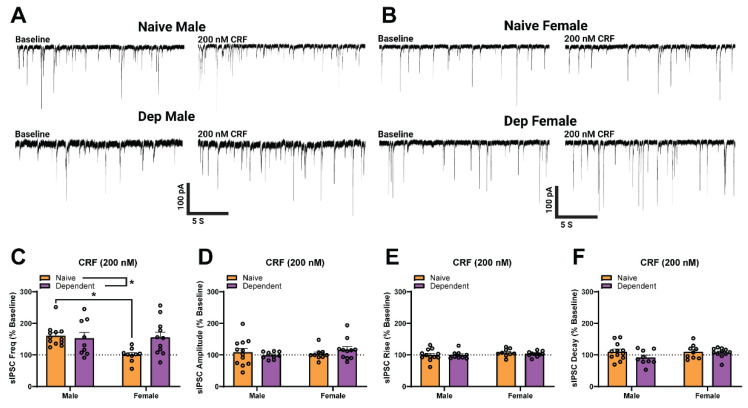
GABAergic transmission in the central amygdala (CeA) of naïve females is insensitive to corticotropin releasing factor (CRF) compared to males. (**A**,**B**) Representative GABA_A_-mediated sIPSCs from CeA neurons of naïve (**upper**) and dependent (**lower**) male (**A**) and female (**B**) rats at baseline (**left**) and during acute application of CRF (200 nM; **right**). (**C**) Acute CRF increases CeA sIPSC frequency in naïve (160.4 ± 9.8% of baseline) and dependent (157.1 ± 18.2% of baseline) males. Acute CRF has no effect on sIPSC frequency in naïve (100.3 ± 7.3% of baseline) females but increases sIPSC frequency in dependent (155.0 ± 16.9% of baseline) females. For sIPSC frequency, the main effect of Alcohol Exposure was observed, as well as a significant interaction effect between Sex × Alcohol Exposure but no main effect of Sex. There were no significant differences in sIPSC amplitude (**D**), rise time (**E**), or decay time (**F**) during acute CRF application across groups. Differences in sIPSC properties were assessed using two-way ANOVA test with a post-hoc Šídák’s correction for multiple comparisons where * denotes *p* < 0.05. Bars represent Mean ± SEM; *n* = 9–12 neurons per group; *N* = 5–8 rats per group.

We next investigated the effects of acute CRF on CeA sIPSCs of both sexes (Figure 2A,B), at a concentration previously determined to have a maximal effect in males (200 nM, [17]). We found a significant main effect of Alcohol Exposure on sIPSC frequency (two-way ANOVA; *F*_1,37_ = 5.01, *p* < 0.05; Figure 2C) such that in alcohol-dependent groups, acute application of CRF significantly increased the sIPSC frequency (as a percent of baseline; see Table A1 in Appendix A for raw values) to a larger extent than in naïve groups. While we did not find a significant main effect of sex, there was a significant Sex × Alcohol Exposure interaction effect (two-way ANOVA; *F*_1,37_ = 4.37, *p* < 0.05) driven by naïve females. Specifically, acute application of 200 nM CRF did not significantly increase the sIPSC frequency of naïve females as it did in naïve males (Šídák; *t*_9,12_ = 3.09, *p* < 0.05). There were no significant differences in sIPSC amplitude (Figure 2D), rise time (Figure 2E), or decay time (Figure 2F) across groups. These results indicate that acute CRF enhances presynaptic GABA release in all groups except naïve females and that alcohol dependence induces similar responsivity to acute CRF in males and females.

**Figure 3:**
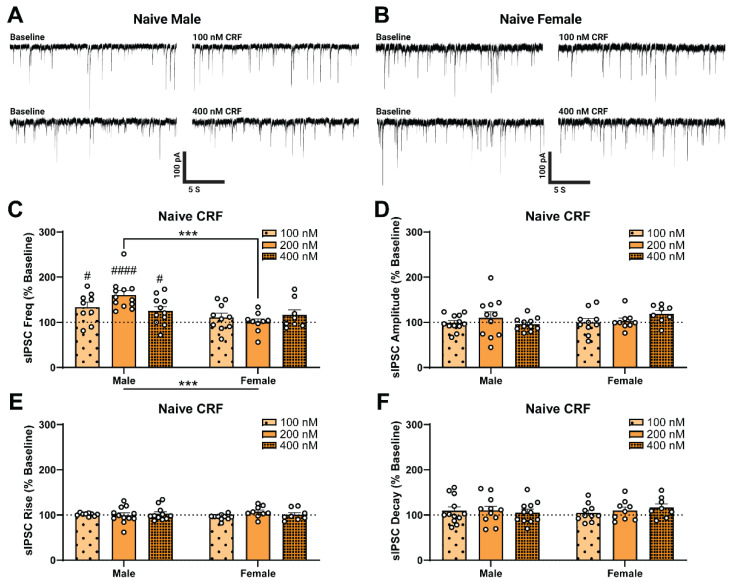
Spontaneous GABAergic transmission in the central amygdala (CeA) of naïve females is insensitive to corticotropin releasing factor (CRF). (**A**–**F**) Male and female sIPSC responses to varied concentrations of CRF. Representative GABA_A_-mediated sIPSCs from CeA neurons of naïve male (**A**) and female (**B**) rats at baseline (**left**) and during acute application of 100 nM CRF (**right**, **upper**) or 400 nM CRF (**right**, **lower**). (**C**) While CRF significantly increases sIPSC frequency above baseline in males, CRF has no significant effect on naïve females. In addition to a main effect of Sex, we observed a Sex × CRF Concentration interaction effect, but no main effect of CRF Concentration. There were no main effects of Sex or CRF Concentration and no interaction effect on sIPSC amplitude (**D**), rise time (**E**), or decay time (**F**). Changes from baseline sIPSC properties were assessed using one-sample *t*-tests where # denotes *p* < 0.05 and #### denotes *p* < 0.0001. Differences between naïve male and female sIPSC properties in response to CRF Concentrations were assessed using two-way ANOVA test with a post-hoc Šídák’s correction for multiple comparisons where *** denotes *p* < 0.001. Bars represent Mean ± SEM; 200 nM responses are the same as in Figure 2; *n* = 8–12 neurons per group; *N* = 4–7 rats per group.

Given that the sex difference in CRF response was driven by naïve females, we tested lower (100 nM) and higher (400 nM) concentrations of CRF in naïve rats of each sex to compare their responsivity (Figure 3A,B). As shown in Figure 3C, we found the main effect of Sex (two-way ANOVA; *F*_1,53_ = 14.34, *p* < 0.001), but no main effect of CRF concentration on sIPSC frequency (as a percent of baseline; see Table A1 in Appendix A for raw values). However, we do report a Sex × CRF Concentration interaction effect (*F*_1,53_ = 3.73, *p* < 0.05) which was driven by the difference between male and female naïve responses to 200 nM CRF (Sidak; *t*_12,9_ = 4.45, *p* < 0.001). In addition, naïve males responded to low (one-sample *t*-test, *t*_9_ = 3.06, *p* < 0.05) and high (one-sample t-test, *t*_11_ = 2.69, *p* < 0.05) concentrations of CRF, with a maximally effective concentration of 200 nM (one-sample *t*-test, *t*_12_ = 6.14, *p* < 0.0001), which recapitulates our previous work. In contrast, we observed no concentration of CRF that produced an effective response in females (one-sample *t*-test, *p* > 0.05). Collectively, these results suggest that acute CRF produces concentration-dependent responses in naïve males but not in females. There were no effects of Sex or CRF Concentration on sIPSC amplitude (Figure 3D), rise time (Figure 3E), or decay time (Figure 3F).

**Figure 4:**
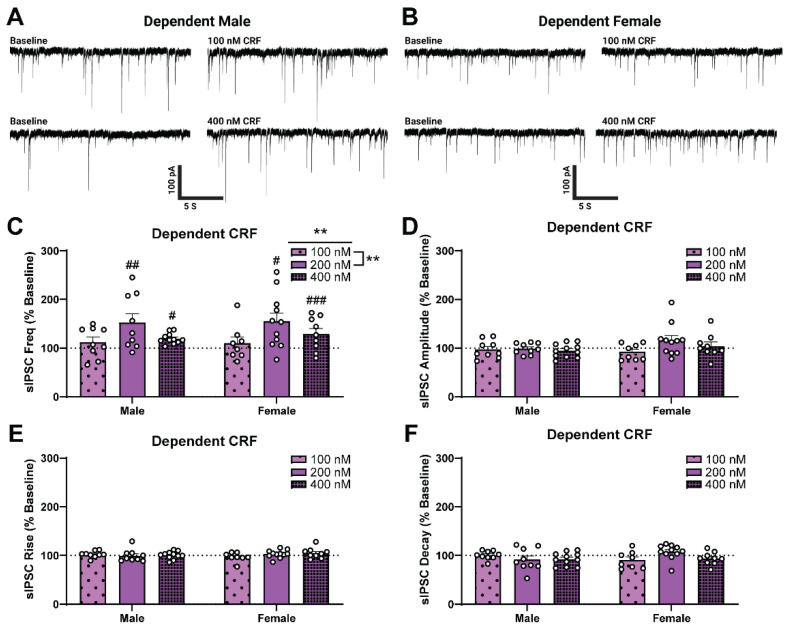
Alcohol dependence induces corticotropin releasing factor (CRF) responsivity of GABAergic synapses in female central amygdala (CeA). (**A**,**B**) Representative GABA_A_-mediated sIPSCs from CeA neurons of dependent male (**A**) and female (**B**) rats at baseline (**left**) and during acute application of 100 nM CRF (**right**, **upper**) or 400 nM CRF (**right**, **lower**). (**C**–**F**) Alcohol-dependent male and female sIPSC responses to varied CRF Concentrations. There is a main effect of CRF Concentration on sIPSC frequency (**C**), but no main effect of Sex and no interaction effect. There was no main effect of Sex or CRF Concentration and no interaction effect on sIPSC amplitude (**D**), rise time (**E**), or decay time (**F**). Changes from baseline sIPSC properties were assessed using one-sample *t*-tests where # denotes *p* < 0.05, ## denotes *p* < 0.01 and ### denotes *p* < 0.001. Differences between male and female sIPSC properties in response to CRF Concentrations were assessed using two-way ANOVA test with a post-hoc Šídák’s correction for multiple comparisons where ** denotes *p* < 0.01. Bars represent Mean ± SEM; 200 nM responses are the same as in Figure 2; *n* = 8–11 neurons per group; *N* = 3–8 rats per group.

We then assessed the CRF sensitivity of male and female dependent rats (Figure 4A,B). Consistent with previous findings [17], high (400 nM) concentrations of CRF increased sIPSC frequency of dependent males (one-sample *t*-test, *t*_9_ = 2.52, *p* < 0.05) while a low concentration of CRF (100 nM; one-sample *t*-test, *p* > 0.05) did not. In addition, high (400 nM) but not low (100 nM) concentrations of CRF also increased sIPSC frequency of dependent females (one-sample *t*-test, *t*_11_ = 5.99, *p* < 0.001), similar to what we observed in the dependent males (Figure 4C). In contrast to the naïve group, group analysis of dependent rats revealed a significant main effect of CRF Concentration (two-way ANOVA test, *F*_2,51_ = 6.19, *p* < 0.01) on sIPSC frequency (as a percent of baseline; see Table A1 in Appendix A for raw values), but no main effect of Sex and no interaction effect. Additionally, there was no main effect of Sex, no main effect of CRF Concentration, and no interaction effect on sIPSC amplitude (Figure 4D), rise time (Figure 4E), or decay time (Figure 4F). These results indicate that in alcohol dependence, the female CeA becomes responsive to acute CRF, and the CRF concentration responsivity is similar to dependent males.

### 2.3. Alcohol Dependence Alters CRF_1_ Receptor Expression in Females

**Figure 5:**
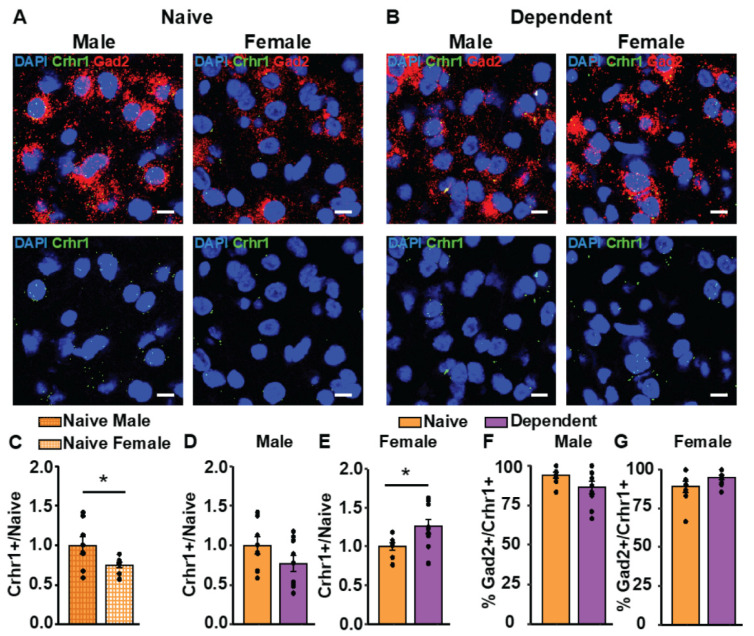
Alcohol dependence alters *Crhr1+* cells in the central amygdala (CeA) of females. Representative images of *Crhr1* (green), *Gad2* (red), and DAPI (blue) for (**A**) naïve and (**B**) alcohol-dependent male (**left**) and female (**right**) rats in the CeA. Scale bar = 10 μm. (**C***–***E**) Summary bar graphs indicating relative proportion of nuclei expressing *Crhr1* (*Crhr1*+) between (**C**) naïve male and female rats, (**D**) naïve male and dependent male rats, and (**E**) naïve female and dependent female rats. (**F**,**G**) Summary bar graphs indicating the percentage of CeA nuclei co-expressing *Gad2* in the *Crhr1*+ population (*Gad2*+/*Crhr1*+) in naïve and dependent male (**F**) and female (**G**) rats. *n* = 7–9 images from *N* = 3 rats/group; bars represent Mean ± SEM; * denotes *p* < 0.05.

CRF_1_ receptors mediate most of the effects of CRF on GABA signaling within the CeA [17,31,32]. Given that the CRF responses of GABAergic synapses varied by sex, we investigated whether these differences reflected changes in CRF_1_ receptor expression in GABAergic neurons in the CeA. To address this, we utilized in situ hybridization (RNAscope) to identify the percent of nuclei expressing CRF_1_ (*Crhr1+*), and GAD2 (*Gad2+*) mRNA in CeA sections of naïve (Figure 5A) and alcohol-dependent (Figure 5B) female and male rats. First, we analyzed the basal CeA expression patterns of *Crhr1* in naïve rats of each sex and found that females have significantly fewer *Crhr1*+ cells relative to males (Figure 5C; *p* < 0.05). We then determined the expression pattern of *Crhr1* in dependent rats. We found that in dependent males, the amount of *Crhr1*+ cells did not significantly differ from naïve males (Figure 5D); however, female dependent rats had significantly more *Crhr1*+ cells than naïve females (Figure 5E; *p* < 0.05). Both female and male rats displayed a high co-expression of *Gad2*+ in the *Crhr1*+ cell population (Figure 5F,G). These data suggest that the CeA of naïve female rats has a lower percentage of *Crhr1*+ cells than naïve males, but alcohol dependence increases the percentage of *Crhr1*+ cells in females.

### 2.4. Alcohol Dependence Induces Tonic Activation of CRF_1_ at CeA GABAergic Synapses in Males Only

**Figure 6:**
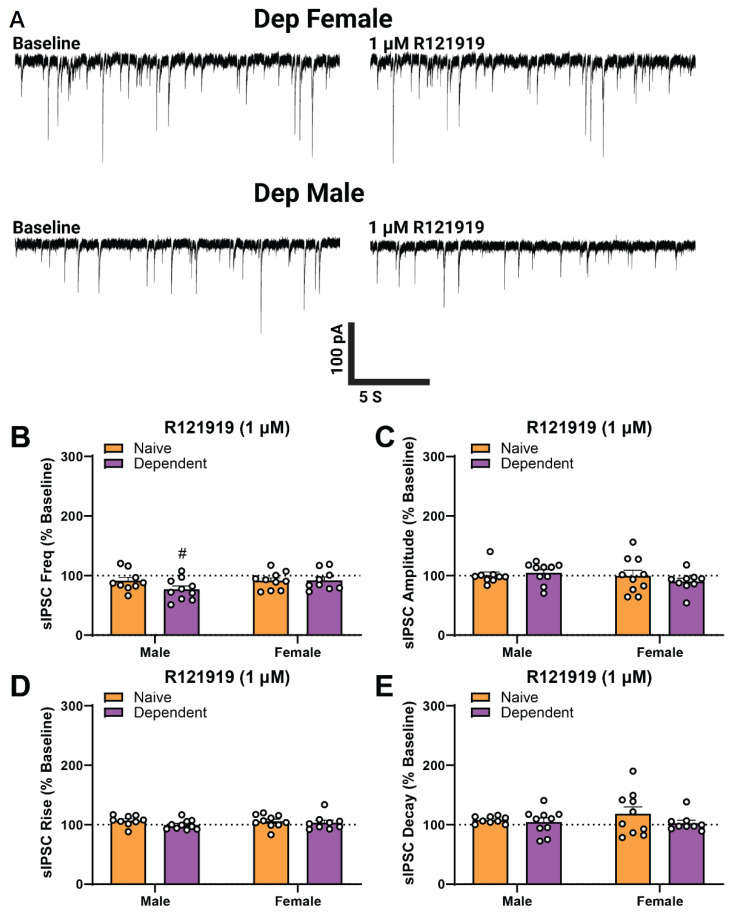
Alcohol-dependent males, but not females, have a basal CRF_1_-mediated tone in the central amygdala (CeA)**.** (**A**) Representative GABA_A_-mediated sIPSCs from CeA neurons of dependent female (**upper**) and male (**lower**) rats at baseline (**left**) and during acute application of R121919 (1 µM; **right**). There was no main effect of Sex or Alcohol Exposure and no interaction effect of R121919 on sIPSC frequency (**B**), amplitude (**C**), rise time (**D**), or decay time (**E**). Changes from baseline sIPSC properties were assessed using one-sample *t*-tests where # denotes *p* < 0.05. Group differences in the sIPSC responses to R121919 were assessed using two-way ANOVA test but were not observed. *n* = 9–10 neurons per group; *N* = 5–6 animals per group; bars represent Mean ± SEM.

Lastly, to compare the basal function of CRF_1_ on CeA GABA transmission in each sex, we tested the effects of CRF_1_ selective antagonism [17,33] on sIPSCs via acute application of 1 µM R121919 (Figure 6A). There was no significant main effect of Sex or Alcohol Exposure and no interaction effect on sIPSC frequency (Figure 6B) amplitude (Figure 6C), rise time (Figure 6D), or decay time (Figure 6E). While selective antagonism of CRF_1_ did not alter CeA GABAergic transmission in naïve males, it significantly decreased GABA release from baseline in dependent male rats (Figure 6B; one-sample *t*-test, *t*_9_ = 2.971, *p* < 0.05). In contrast to the males, R121919 did not alter sIPSC properties (as a percent of baseline; see Table A1 in Appendix A for raw values) in either naïve or dependent female rats, suggesting that tonic activation of CRF_1_ may be specific to dependent males.

## 3. Discussion

**Figure 7:**
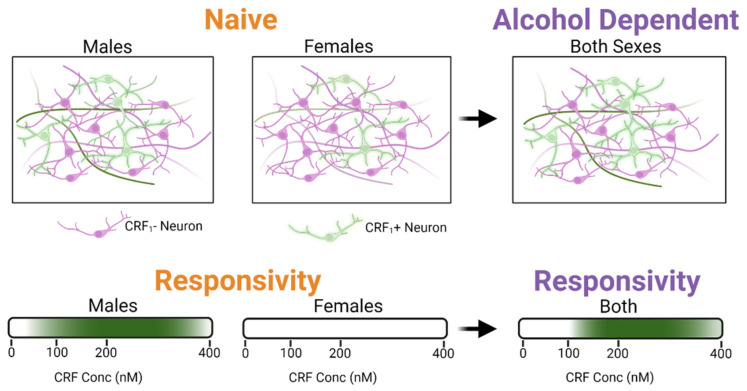
Summary of synaptic changes observed in the central amygdala (CeA) during alcohol dependence in male and female rats. All neurons represented in the top figure are GABAergic. Green denotes CRF_1_+ neurons, while purple denotes CRF_1_− neurons. Naïve females have lower CRF_1_ expression on GABAergic neurons and are less responsive to corticotropin releasing factor (CRF) than naïve males. In alcohol-dependent females, CRF_1_ expressing cell populations and CRF responsivity increase, similar to levels observed in dependent males. Created with BioRender.com.

In this study, we identified distinct, sexually dimorphic responses of the CRF system on GABAergic synapses in the CeA of naïve and alcohol-dependent rats (see schematic in Figure 7). In brief, we found that CeA neurons of naïve male and female rats display similar baseline presynaptic GABAergic inputs and postsynaptic receptor function. However, alcohol dependence induced an increase in the baseline decay times of postsynaptic GABA_A_ receptor-mediated currents in CeA neurons of both sexes. We recapitulated our previous work [17,34,35] showing that a maximal concentration (200 nM) of CRF increased action-potential dependent GABA release in the CeA of naïve and dependent male rats but found naïve female CeA unresponsive to CRF. We then characterized the effects of high and low CRF concentrations in each sex, which confirmed that naïve females do not respond to CRF. Interestingly, alcohol dependence induced CRF responsivity in females to a similar degree observed in dependent males such that no sex difference was observed. We hypothesized that this heightened responsivity to CRF was due to changes in CRF_1_ expression in GABAergic neurons in the CeA, which was confirmed via in situ hybridization. Lastly, selective antagonism of CRF_1_ with R121919 revealed that tonic CRF_1_ activation, which regulates GABA release, occurred only in dependent males. R121919 had no effect on naïve or dependent females suggesting a lack of tonic CRF signaling in females.

We recently reported the acute alcohol insensitivity of GABAergic CeA neurons in naïve females, which was maintained in alcohol-dependent females except at the highest concentration (88 mM) of ethanol [30]. Here, we found a similar profile in the CRF system such that naïve female CeA neurons were unresponsive to acute CRF, and alcohol dependence induced responsivity in these CeA neurons to high but not low concentrations of CRF. Other studies have reported similar sexually dimorphic effects of the CRF system in the CeA. For example, Rouzer et al. found a Sex × Age interaction in basal spontaneous GABA synaptic transmission within the rat CeA, but only age differences in action potential-independent GABA release [36]. However, sex differences mediated by the selective CRF_1_ agonist Stressin-1 emerged: opposing effects of Stressin-1 were observed in the action potential-independent GABA release of adolescent and adult males, while in the females there was no change in the response between adolescents and adults [36]. Furthermore, in a study using transgenic CRF_1_ reporter mice, voluntary alcohol drinking increased the sensitivity of CRF_1_-positive neurons in the CeA to the effects of acute alcohol in males but not females. In contrast, alcohol drinking increased acute CRF sensitivity of these neurons in both males and females [37]. Tonic CRF activity was also found to be sexually dimorphic, as the CRF_1_ antagonist R121919 decreased GABA release in water and alcohol-drinking females, but not in either male group [37]. A study by Retson and colleagues also reported sex differences in the CRF system of the rat CeA, which found that alcohol drinking activated CeA CRF neurons and enhanced the response of these neurons to stress selectively in male but not female rats [38].

Here, we found that alcohol dependence increased the CRF responsivity of GABAergic synapses in females while male responses remained elevated. Our results also showed that CRF_1_ antagonism using R121919 significantly decreased (action-potential dependent) GABA release in dependent males, revealing a basal activity of these receptors in the modulation of CeA GABA transmission after chronic ethanol exposure. In contrast, CRF_1_ antagonism did not alter basal CeA GABA transmission in either naïve or dependent female rats. A similar difference was seen for action-potential independent GABA release by Rouzer et al., where the CRF_1_ selective antagonist NBI 35965 increased GABA release in adult male but not female rats [36]. To that end, some limitations of the current study are that we only investigated action-potential dependent GABA transmission in the CeA of adult rats. As such, the role of age in the sexually distinct responses of the CRF system during alcohol dependence remains unclear. Furthermore, our conclusions may not apply to action-potential independent GABA transmission, which are important issues to be addressed in future work. Additionally, given that we found that dependence increased baseline sIPSC decay times without affecting frequency or amplitude, we also assessed the charge transfer (or integrated area) of sIPSCs, but found no differences in inhibitory synaptic activity. Some potential mechanisms underlying this phenomenon include a change in GABA subunit expression [39,40] or a reduction in the expression of a GABA transporter that removes GABA from the synaptic space [41]. Such work is outside the scope of the current study but would be an important area for future investigation.

Some of the discrepancies across studies may be explained by the differential effects produced by alcohol paradigms (i.e., voluntary alcohol drinking vs. vapor-induced alcohol dependence) and the age of the study animals. It is also important to be cautious when drawing comparisons between studies using the CRF peptide and CRF_1_-selective agonists, as such compounds may produce distinct synaptic responses. That is, CRF as a non-selective agonist can affect both CRF_1_ and CRF_2_ receptors, producing changes in synaptic transmission that may not be identical to those induced by the selective CRF_1_ activation used by Rouzer et al. [36]. However, another important factor to consider is the composition of the cell population being studied, which can be associated with distinct responses. For instance, the Agoglia et al. study specifically targeted CRF_1_+ neurons in mice [37], while our electrophysiology data come from neurons of unknown CRF_1_ expression in rats. As our in situ hybridization data show, naïve females have fewer GABAergic neurons expressing CRF_1_. Thus, our selection of neurons in females was less likely to be CRF_1_+ than in males. It is possible that the sensitivity of rat CeA CRF_1_+ neurons to CRF system agonism and antagonism matches that seen in mice, although re-examination of these effects in labeled CRF_1_+ neurons of rats would need to be performed in future studies to be certain.

However, in light of recent work [42], another possibility is that the animal models used (rats vs. mice) could be responsible for these differences. The alcohol-induced increase in GABA release upon acute CRF application in the CeA is an effect we previously demonstrated in many species including mice, rats, and non-human primates [10,17,31,32,43]. In rats, this increase in GABA in the CeA was implicated in excessive alcohol drinking of dependent animals, and this effect is mediated by locally projecting CRF neurons in the CeA [44,45,46]. However, in mice, chemogenetic activation or inhibition of these local CeA CRF neurons had no effect on alcohol consumption [42], whereas modulation of BNST CRF neurons suppressed binge drinking [47]. Thus, the involvement of the CeA CRF system in alcohol dependence may be species-dependent, and this difference in CRF system functioning could explain the discrepancies between the rat and mouse data, including the dichotomous sex differences.

Overall, our findings provide insight into the function of the CRF/CRF_1_ system on GABAergic transmission in the CeA of females and identify maladaptations in this system that occur during alcohol dependence. More work is needed to provide a greater overall insight into the mechanisms by which the CRF system modulates excitatory–inhibitory balance within the CeA, as this system has yet to be studied in the context of female glutamatergic transmission [33].

## 4. Materials and Methods

### 4.1. Animals

Female (*N* = 45) and male (*N* = 40) Sprague Dawley rats (Charles River, Raleigh, NC, USA) weighed on average 257.2 ± 9.3 g and 354.85 ± 16.9 g, respectively, at time of sacrifice. Estrous cycle was determined before euthanasia, but not selected for. All rats were housed in a temperature- and humidity-controlled room on a 12 h light/dark cycle with food and water available ad libitum. Alcohol-dependent rats (average blood alcohol level 172.68 ± 17.4 mg/dL at time of sacrifice) were generated by exposure to alcohol vapor daily (14 h alcohol vapor; 10 h air vapor) for 5–7 weeks [30,48,49,50] Female (*N* = 6) and male (*N* = 6) Wistar rats (Charles River, Raleigh, NC, USA) weighed on average 253.17 ± 12.7 g and 387.67 ± 24.5 g, respectively, at time of sacrifice. All procedures and care were conducted in accordance with the National Institutes of Health Guide for the Care and Use of Laboratory Animals and were reviewed and approved by the Institutional Animal Care and Use Committee of The Scripps Research Institute.

### 4.2. Electrophysiology

Preparation of acute brain slices and electrophysiological recordings were performed as previously described [17,30,51,52]. Rats were deeply anesthetized with isoflurane (3–5%) before decapitation and brain isolation. Coronal CeA slices (300 μm) were prepared using a Leica VT1200 vibratome (Leica Biosystems, Deer Park, IL, USA) in an ice-cold high-sucrose cutting solution (sucrose 206 mM; KCl 2.5 mM; CaCl_2_•2H_2_O 0.5 mM; MgCl_2_ 7 mM; NaH_2_PO_4_ 1.2 mM; NaHCO_3_ 26 mM; glucose 5 mM; HEPES 5 mM; Sigma-Aldrich, St. Louis, MO, USA). Slices were incubated and superfused (flow rate of 2–4 mL/min) with carbogen (95% O_2_/5% CO_2_) equilibrated artificial cerebrospinal fluid (aCSF; in mM: 130 NaCl, 24 NaHCO_3_, 10 glucose, 1.5 MgSO_4_•7H_2_O, 3.5 KCl, 1.25 NaH_2_PO_4_•H_2_O, 2 CaCl_2_•2H_2_O; Sigma-Aldrich, St. Louis, MO, USA). Whole-cell patch-clamp recordings of GABAergic spontaneous inhibitory postsynaptic currents (sIPSCs) were performed in neurons from the medial subdivision of the CeA clamped at −60 mV. Patch pipettes (3 to 6 MΩ) were filled with an internal solution composed of (in mM; Sigma-Aldrich, St. Louis, MO, USA): 145 KCl, 0.5 EGTA, 2 MgCl_2_, 10 HEPES, 2 Mg-ATP, and 0.2 Na-GTP. For animal variability, each experimental group contained neurons from a minimum of 3–4 rats. GABAergic activity was pharmacologically isolated with 20 μM 6,7-dinitroquinoxaline-2,3-dione (DNQX), 30 μM DL-2-amino-5-phosphonovalerate (DL-AP5), and 1 μM CGP 55845A. In all experiments, cells with a series resistance greater than 20 MΩ were excluded from analysis, and series resistance was periodically monitored during gap-free recording with a 10 mV pulse. Cells in which series resistance changed more than 25% during the experiment were excluded from analysis. Data were analyzed using Mini Analysis (Synaptosoft Inc., Fort Lee, NJ, USA) with 3-min bins of gap-free recording. All drugs were applied by bath superfusion.

### 4.3. Drugs

CRF, CGP 55845A, DL-AP5, and DNQX were obtained from Tocris (Ellisville, MO, USA). Drugs were added to the aCSF from stock solutions to obtain known concentrations in the superfusate. Stock solutions of AP-5, CGP 55845A and CRF were prepared in distilled water, while DNQX and R121919 hydrochloride (R12) were dissolved in 100% DMSO. All drugs were applied to the bath solution to achieve the final desired concentrations. The final DMSO concentration in the bath solution did not exceed 0.15%.

### 4.4. In Situ Hybridization and Confocal Microscopy

Male and female Wistar rats (3 per treatment group) were anesthetized with isoflurane and transcardially perfused with ice-cold phosphate-buffered saline (PBS) followed by Z-fix (Fisher Scientific, Waltham, MA, USA). Brains were dissected, immersion fixed in Z-fix at 4 °C for 24 h, cryoprotected in 30% sucrose in PBS at 4 °C for 24–48 h, flash frozen in isopentane on dry ice, and stored at −80° C. Brains were then sliced on a cryostat into 20 µm thick sections, mounted on SuperFrost Plus slides (Fisher Scientific, Waltham, MA, USA; Catalog No. 1255015), and stored at −80 °C until use. In situ hybridization was performed using RNAscope fluorescent multiplex kit (ACD Bio-techne, Newark, CA, USA; Catalog No. 320850) as previously described [53]. Briefly, target retrieval pretreatment was performed according to the RNAscope manual, where slides were submerged at 95–98 °C for 10 min in target retrieval buffer (ACD, Catalog No. 322000), immediately rinsed in distilled water, and then dehydrated in ethanol (stored at −80 °C if needed), followed by incubation at 40 °C for 20 min with protease IV. Next, the RNAScope Fluorescent Multiplex Reagent Kit User Manual was followed exactly. Lastly, slides were mounted and coverslipped with Vectashield + DAPI (Fisher Scientific, Fair Lawn Industrial Park, NJ, USA). The probes used from ACD Bio-techne were as follows: 3-plex negative control (ACD, Catalog No. 320871), *Crhr1* (ACD, Catalog No. 318911-C1), and *Gad2* (ACD, Catalog No. 435801-C2).

Images of the CeA were acquired with a Zeiss LSM 780 laser scanning confocal microscope (40X oil immersion, 1024 × 1024 pixel, 5 μm z-stacks). All microscope settings were kept the same within experiments. Quantification was performed with the image analysis software CellProfiler [54] using the recommended guidelines for analysis of RNAscope images by ACD Biotechne with background (negative control) subtraction. Nuclei were considered positive if they contained one or more puncta after thresholding out any background. Images were visually inspected for accuracy if required. The percent of positive nuclei was calculated for each probe, and in instances of multiple treatment group comparisons, data were normalized to the control/naïve group to show relative values. Outliers were detected with Grubb’s test. Analysis was performed on raw images, and brightness/contrast/pixel dilation are the same for all representative images shown per figure.

### 4.5. Statistical Analysis

Data are presented as Mean ± SEM of either raw values or were normalized to the baseline values, and *n* refers to the number of cells, while *N* refers to the number of used rats (exact values are indicated for each experiment). To avoid pseudo-replication (i.e., collecting multiple samples from an individual animal), no more than 3 data points were collected from any single animal. The criterion for statistical significance was *p* < 0.05. Statistical analyses were performed in Prism 9 (GraphPad Software, La Jolla, CA, USA). Data were analyzed with two-way ANOVA (Alcohol Exposure × Sex or Sex × CRF Concentration) followed by Šídák’s post-hoc comparisons (when appropriate.) Changes over baseline (e.g., agonist/drug responses) were assessed using one-sample *t*-tests where indicated. 

## Data Availability

The data underlying this article will be shared upon reasonable request to the corresponding author.

## Appendix A

**Table A1:** Raw value group averages of each spontaneous inhibitory postsynaptic current (sIPSC) parameter tested.

Alcohol Exposure	Sex	sIPSC Property	Baseline		100 nM CRF		Baseline		200 nM CRF		Baseline		400 nM CRF		Baseline		R121919	
			Mean	SEM	Mean	SEM	Mean	SEM	Mean	SEM	Mean	SEM	Mean	SEM	Mean	SEM	Mean	SEM
Naïve	Male	Frequency (Hz)	2.99	0.68	3.93	0.99	1.13	0.23	1.85	0.38	3.14	0.90	3.63	1.02	3.16	0.77	2.86	0.68
		Amplitude (pA)	62.98	7.63	61.77	6.76	70.07	10.07	66.88	6.86	59.91	6.25	56.49	5.68	79.49	9.00	81.41	10.59
		Rise Time (ms)	2.57	0.13	2.59	0.11	2.61	0.14	2.54	0.10	2.55	0.08	2.61	0.12	2.33	0.08	2.49	0.11
		Decay Time (ms)	9.46	1.16	9.68	0.84	9.68	0.84	8.23	1.04	9.41	0.85	9.61	0.80	10.28	1.27	11.32	1.43
		*n*	9				12				11				9			
	Female	Frequency (Hz)	1.48	0.36	1.52	0.28	1.73	0.58	1.61	0.49	1.52	0.34	1.94	0.58	2.25	0.58	2.01	0.51
		Amplitude (pA)	66.42	5.97	64.47	6.52	65.71	5.13	66.58	4.94	57.95	5.77	69.99	9.01	68.21	6.96	65.28	6.99
		Rise Time (ms)	2.73	0.11	2.60	0.11	2.04	0.11	2.19	0.15	2.65	0.12	2.65	0.10	2.84	0.16	3.01	0.19
		Decay Time (ms)	7.47	0.82	7.77	0.87	5.92	0.81	6.57	1.03	7.76	1.27	8.74	1.19	9.95	0.98	11.30	1.07
		*n*	10				9				8				10			
Dependent	Male	Frequency (Hz)	3.30	0.40	3.56	0.47	1.43	0.29	2.25	0.68	2.63	0.54	3.14	0.68	1.79	0.42	1.48	0.37
		Amplitude (pA)	85.01	10.06	83.44	13.25	61.75	7.82	62.05	7.78	80.04	10.23	78.06	12.82	57.55	6.56	59.45	6.47
		Rise Time (ms)	2.42	0.06	2.48	0.06	2.63	0.09	2.61	0.10	2.56	0.07	2.56	0.06	3.14	0.16	3.09	0.18
		Decay Time (ms)	10.20	1.03	10.16	0.92	9.71	1.44	8.87	1.28	10.61	0.72	9.91	0.87	12.68	1.20	13.04	1.02
		*n*	9				9				11				10			
	Female	Frequency (Hz)	2.42	0.56	2.42	0.56	1.50	0.21	2.57	0.57	1.63	0.23	2.13	0.37	1.93	0.62	1.68	0.48
		Amplitude (pA)	59.18	3.40	53.49	2.23	95.01	15.07	112.62	21.57	62.84	8.16	68.50	15.50	71.30	12.03	62.58	9.93
		Rise Time (ms)	2.82	0.25	2.73	0.27	2.23	0.09	2.29	0.07	2.61	0.19	2.72	0.20	3.10	0.18	3.19	0.16
		Decay Time (ms)	12.09	1.15	10.84	1.17	8.45	0.88	9.15	1.11	10.81	0.86	10.14	0.95	11.47	1.28	11.70	1.14
		*n*	8				11				9				9			
